# Peritoneal Carcinomatosis: When Everything Is Not What It Seems

**DOI:** 10.7759/cureus.52924

**Published:** 2024-01-25

**Authors:** Sofia Perdigão, Rita Cunha, Catarina Costa, Cristiana Sousa, Antonio Teira

**Affiliations:** 1 Internal Medicine, Centro Hospitalar de Trás-os-Montes e Alto Douro, Chaves, PRT; 2 Internal Medicine, Centro Hospitalar Universitário do Algarve, Faro, PRT; 3 Oncology, Centro Hospitalar de Tras-os-Montes e Alto Douro, Chaves, PRT

**Keywords:** ascites, peritoneum, serous membrane neoplasm, malignant peritoneal mesothelioma, peritoneal carcinomatosis

## Abstract

Malignant peritoneal mesothelioma (MPM) is a rare neoplasm with a low incidence rate worldwide but high morbidity and mortality rates. Due to its rarity, the studies are scarce. We present a case of a 73-year-old woman admitted to the internal medicine unit with constitutional syndrome, abdominal pain, and ascites. Throughout the investigation, aspects suggestive of peritoneal carcinomatosis were identified. An extensive study was then carried out in an attempt to identify the primary tumor, which proved to be unsuccessful. During the two weeks of hospitalization, the patient’s clinical condition worsened, with an increase in ascites and a deterioration in her general health. This case was then discussed with an oncology consultant, and it was decided to biopsy a peritoneal implant with the support of interventional radiology. MPM was then diagnosed through histopathology. With this case, the authors intend to highlight that, although rare, this diagnosis should be considered when appropriate and that even in the suspicion of secondary disease, the primary tumor should always be identified, as localized MPM may be curable.

## Introduction

Malignant mesothelioma is a serous membrane neoplasm with a high fatality rate. It is difficult to diagnose because of its rarity and because symptoms are not specific, determining that a high suspicion level must be involved to equate this diagnosis. It can involve any of the serous membranes: pleura, pericardium, peritoneum, and tunica vaginalis. It is a rare disease, with peritoneal mesothelioma being the second most common, following that of the pleura. Studies are scarce and outdated due to the rarity of the disease but the incidence suggests there are 3,300 cases per year in the USA, of which only 10% to 15% are peritoneal mesotheliomas [1-3]. Although the main risk factor is exposure to pollutants, in particular, asbestos, some specific genes and mutations in molecular pathogenesis have been documented [4-6]. It is known that, with regard to malignant peritoneal mesothelioma (MPM), exposure to asbestos is a cumulative risk factor. Regarding distribution by sex, women comprise approximately one-third to one-half of all cases of MPM [7]. It is also known to be a disease with a long delay in diagnosis, which is why it is important to keep this disease in mind.

## Case presentation

A 73-year-old woman presented to the emergency department with constitutional syndrome, abdominal pain, and diarrhea. She had a history of hypertension, type 2 diabetes, dyslipidemia, pancreatic cysts, and fibromyalgia, with no risk of exposure to pollutants. The patient had already undergone a pancreatic echo-endoscopy with a biopsy that documented a serous cyst. Prior to admission, she underwent an upper and lower digestive endoscopy, and serum tumor markers were normal.

**Figure 1:**
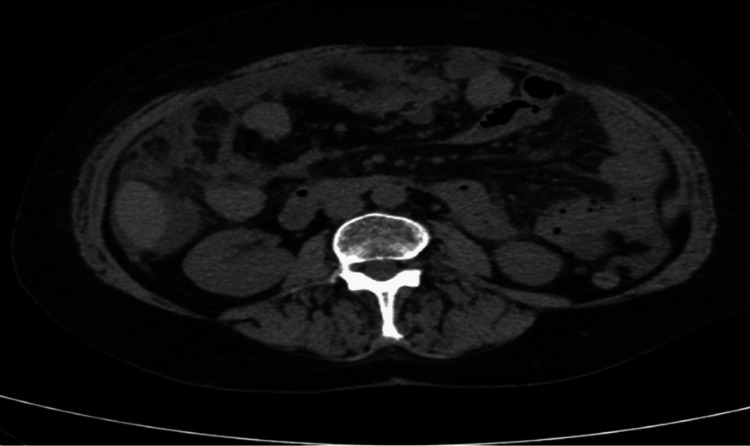
Computed tomography of the abdomen revealed densification of the omentum, suggesting peritoneal carcinomatosis and secondary involvement.

**Figure 2:**
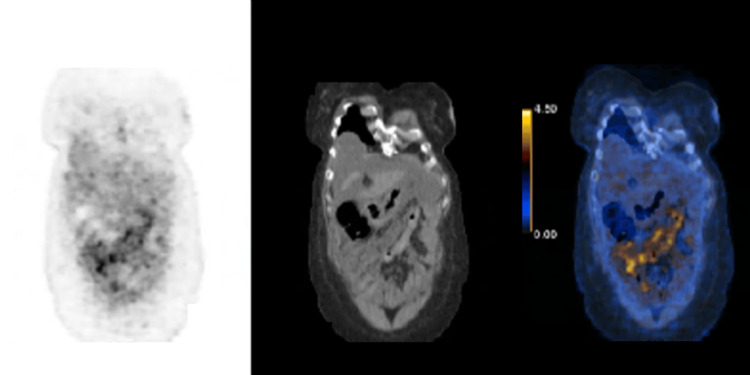
18F-fluorodeoxyglucose (FDG) positron emission tomography scan revealed extensive diffuse hypermetabolic peritoneal densifications.

During her hospital stay, she was diagnosed with abdominal ascites. She also presented with an increase in abdominal pain, for which a computed tomography (CT) of the thorax, abdomen, and pelvis was carried out, revealing peritoneal effusion with signs of peritoneal carcinomatosis, especially in the lesser omentum, and edema of the adnexa, suggesting secondary involvement (Figure 1). The ascites was not accessible via paracentesis, so a diagnostic paracentesis was not performed. A breast ultrasound was performed, which revealed only non-pathological microcalcifications and an abdominal ultrasound confirmed aspects suggestive of peritoneal carcinomatosis. Because the primary tumor was not yet identified, the patient underwent an 18F-fluorodeoxyglucose (FDG) positron emission tomography (PET) scan. The exam revealed hypermetabolic abdominal-pelvic adenopathies and extensive diffuse hypermetabolic peritoneal densifications with peritoneal effusion, suggestive aspects of peritoneal carcinomatosis (Figure 2).

During the investigation, the patient’s clinical condition worsened, with increased ascites, intensification of abdominal pain, weight loss, and general malaise. Observation by gynecology was also requested during hospitalization to rule out a tumor related to the patient’s age and sex, but this yielded no additional information. After consultation with an oncologist, it was decided to biopsy a peritoneal implant with the help of radiology to obtain a histopathology report on the tumor.

**Figure 3:**
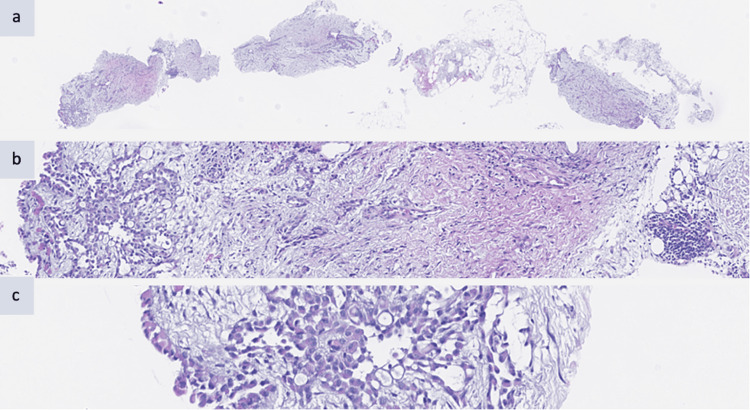
Hematoxylin and eosin (magnification/20x/40x). (a) Biopsy fragment of peritoneum. (b) Fragment demonstrating infiltrative proliferation of the mesothelial epithelioid cells. (c) Mesothelial epithelioid cells with abundant eosinophilic cytoplasm and ovoid nuclei.

**Figure 4:**
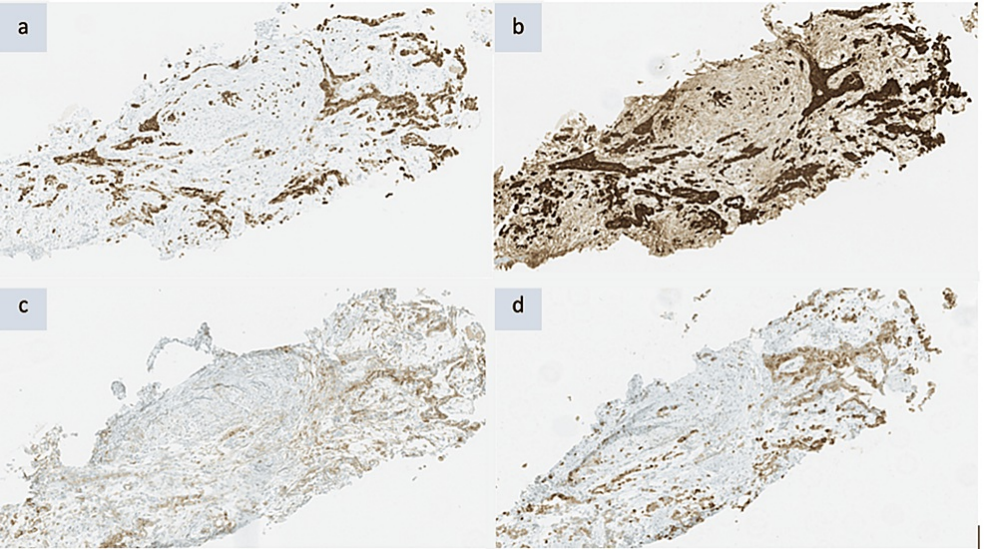
Immunohistochemistry of the biopsy. Immunohistochemical study revealed mesothelial phenotype. (a) Expression of CK7 in the epithelioid cell population. (b) Expression of calretinin in the epithelioid cell population. (c) Expression of D2-40 in the epithelioid cell population. (d) Expression of CK5/6 in the epithelioid cell population.

The biopsy was carried out and the patient went home. Upon discharge, she was prescribed potent analgesics and forwarded very urgently to a group oncology consultation while awaiting the results of the biopsy. The results of the biopsy revealed MPM of the epidermoid type (Figures 3, 4).

Following the results, the patient was observed in ambulatory consultation by an oncologist and subsequently referred to the Portuguese Institute of Oncology (IPO). She then underwent surgery with cytoreduction of the tumor (resection of round and falciform ligament, and resection of omental cake/partial resection of the greater omentum) and hyperthermic intraperitoneal chemotherapy (HIPEC). Due to the overall extension of the disease, the patient was then referred to palliative chemotherapy at her local hospital, which she continues to receive to this day, with a partial response to treatment.

## Discussion

In this case, the patient presented with non-specific symptoms at admission. MPM symptoms are predominantly related to ascites and tumor progression in the abdominal cavity. Common complaints include abdominal pain with distention, nausea, anorexia, and weight loss. Gastrointestinal complications such as bowel obstruction are usually a manifestation of advanced disease [8]. This patient presented with all of the above, with the exception of bowel obstruction; however, these are nevertheless non-specific symptoms that are difficult to evaluate. The workup was consistent only with peritoneal carcinomatosis both on the CT and abdominal ultrasound, and no primary tumor was identified. After a wider study that included advanced diagnostic techniques such as 18-FDG PET and in the absence of primary neoplasia, it was not possible to explain the dissociation between the evidence of extensive secondary involvement and the absence of primary neoplasia. The lack of diagnosis also complicated making a medical decision regarding how to approach the disease and which therapies the patient would be eligible for and could benefit from.

Some data suggest that particles named soluble mesothelin-related peptides (SMRPs), a glycoprotein produced by the breakdown of mesothelin expressed in mesothelial cells and overexpressed in cases of malignant mesothelioma, may help in diagnosis, but they do not establish the diagnosis [9]. Cytologic analysis of ascitic fluid has limited diagnostic utility because fluid cytology is often inconclusive with a low yield, making discerning between benign or malignant mesothelioma especially difficult. Therefore, the best way to establish a diagnosis is by histopathology. There are three broad histologic subtypes: epithelioid, sarcomatoid, and biphasic (mixed). The most common, epithelioid malignant mesotheliomas, are composed of cells that resemble normal mesothelial cells [10]. Histological evidence of invasion is the defining parameter in the distinction between malignant and benign mesothelioma.

In this patient, paracentesis could not be performed. Following the extensive investigation carried out, and after a multidisciplinary discussion, it was decided to biopsy a peritoneal implant, a practice not routinely carried out in the normal investigation of occult neoplasia [11,12].

## Conclusions

Malignant mesothelioma is a rare disease with a low incidence worldwide but high morbidity and mortality rates. Due to its nosological rarity, existing studies are scarce. Exposure to asbestos is a significant risk factor but not a necessary one, which was the case for this patient. The genetic component has an important role yet to be explored. Much is unknown about the pathophysiological mechanisms involved and there is still a vast field to explore. With this case, the authors intend to highlight that, although rare, this diagnosis should be considered when appropriate. Even in the presence of evidence of secondary disease, staging should not be established based solely on these data. In the particular case of MPM, it may be a diffuse or localized disease, the latter being potentially curable, giving a completely different outcome and prognosis for the patient. In this case, unfortunately, the patient had diffuse MPM. Even though she underwent surgical removal and HIPEC in an attempt to eliminate the disease, this was not possible due to its extension. The patient remains to this day with palliative chemotherapy.
